# Genome-editing tools for stem cell biology

**DOI:** 10.1038/cddis.2015.167

**Published:** 2015-07-23

**Authors:** E A Vasileva, O U Shuvalov, A V Garabadgiu, G Melino, N A Barlev

**Affiliations:** 1 Institute of Cytology, RAS, Saint-Petersburg, Russia; 2 Technological University, Saint-Petersburg, Russia; 3 MRC Toxicology Unit, Leicester, UK

## Abstract

Human pluripotent stem cells provide a versatile platform for regenerative studies, drug testing and disease modeling. That the expression of only four transcription factors, Oct4, Klf4, Sox2 and c-Myc (OKSM), is sufficient for generation of induced pluripotent stem cells (iPSCs) from differentiated somatic cells has revolutionized the field and also highlighted the importance of OKSM as targets for genome editing. A number of novel genome-editing systems have been developed recently. In this review, we focus on successful applications of several such systems for generation of iPSCs. In particular, we discuss genome-editing systems based on zinc-finger fusion proteins (ZFs), transcription activator-like effectors (TALEs) and an RNA-guided DNA-specific nuclease, Cas9, derived from the bacterial defense system against viruses that utilizes clustered regularly interspaced short palindromic repeats (CRISPR).

## Facts

- Genome editing systems based on zinc-finger fusion proteins (ZFs), transcription activator-like effectors (TALEs) and an RNA-guided DNA-specific nuclease (Cas9) can be successfully used for generation of induced pluripotent stem cells.
- ZF-TFs and TALENs fused with different transcriptional domains can modulate expression of master genes of pluripotency, such as Oct4, Sox2, Klf4 and c-Myc.
- The CRISPR/Cas9 fusion with the histone acetyltransferase domain of p300 can reactivate on its own the epigenetically silenced locus of *Oct4*, which makes this system a very attractive tool for generation of iPSCs.
- As generation of iPSCs requires p53 inactivation, which, in turn, provokes tumorigenesis, it will be interesting to see whether temporal Cas9d-mediated inhibition of p53 downstream targets, but not p53 itself, is sufficient to trigger dedifferentiation without affecting the quality control.

## Open Questions

- One downside of the iPSCs generation process is its low efficacy. In this respect, what will happen when the precision of genome editing systems is combined with the power of small molecule inhibitors that reverse the epigenetic state of differentiated cells?

## Introduction

**Figure 1:**
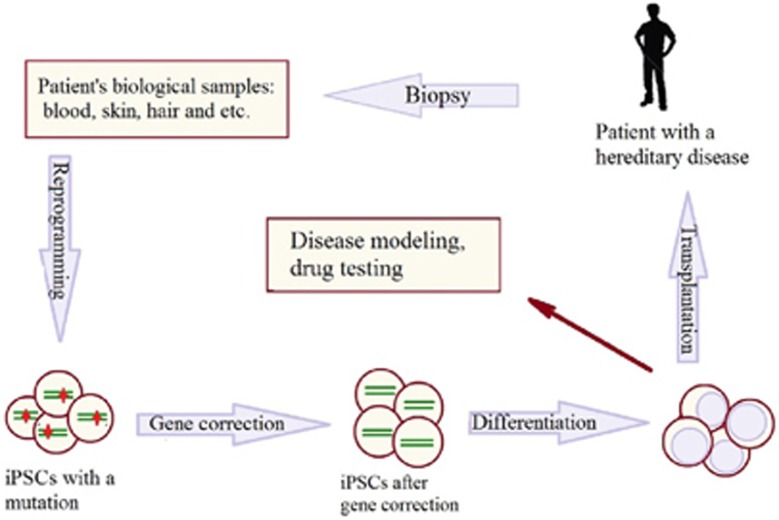
Application of genome editing in molecular medicine (gene therapy, disease modeling). iPCSs could be generated from somatic cells of the patient with monogenic diseases for correction, differentiation into cell types suitable for therapy and transplantation into a patient to restore the function

The emergence of genome-editing technologies over the past several years has flourished the investigation of human cellular disease models. Recent achievements in generation of pluripotent stem cells (PSCs) from patients and specific differentiation of these cells into various somatic cell types greatly facilitated the studies on pathophysiology of socially important diseases (Figure 1). PSCs include embryonic stem cells (ESCs) and induced pluripotent stem cells (iPSCs). PSCs are able to proliferate indefinitely, to self-renew and to develop into more differentiated cell lineages offering the opportunity for human disease modeling^1^ (Figure 1). Pluripotency is characterized by specific configuration of chromatin and epigenetic modifications. Forced expression of four transcription master-regulators, Oct3/4, Sox2, Klf4 and c-Myc (OSKM), were able to overcome the epigenetic traits of differentiated cells and to revert them into the naive pluripotent state.^2^ Both activity and expression of these transcription factors (TFs) are repressed in normal somatic cells and hence their re-activation is instrumental for the re-programming of somatic cells into the iPSCs. The originally described direct delivery of the corresponding cDNAs into somatic cells cannot be utilized for the purpose of gene therapy, because of the possibility of DNA recombination. Another approach is via pharmacological enhancement of the downstream targets of OSKM.^3, 4, 5, 6^ An alternative approach of OSKM re-activation in differentiated cells can be achieved through specific targeting of transcription activators by means of genome editing.

As of today, several efficient systems of genome manipulation have been described based on various classes of DNA-binding chimeric proteins such us zinc-finger proteins (ZFs), transcription activator-like effectors (TALEs) and the guide RNA (gRNA)-driven Cas9d mutant (CRISPR) system. In this review, we discuss the exploitation of various genome editing techniques for successful and robust generation of iPSCs from human somatic cells.

### Systems for genome editing and manipulation of gene expression

#### ZFs nucleases and ZF-TFs

**Figure 2:**
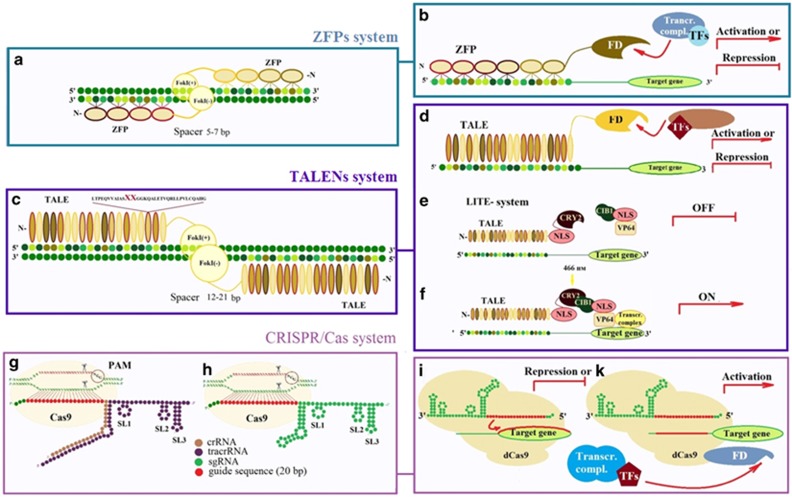
ZFs, TALEs and CRISPR/Cas9 systems for genome editing and gene expression manipulation. ZFP, zinc-finger protein; FD, functional domain; TFs, transcription factors; dCas9, dead Cas9 nuclease; SL1-3, stem loop 1-3; PAM, protospacer adjacent motif. (**a**) Schematic representation of the ZFN (zinc-finger nuclease) system for genome editing. It consists of a zinc-finger DNA-binding domain and a nuclease domain of the FokI endonuclease. (**b**) Site-specific ZF-TFs can either activate or repress gene expression depending on their functional domains (FD). (**c**) Schematic representation of the TALENs system for genome editing. It consists of a TALEs DNA-binding domain and a nuclease domain of the FokI. XX- RVDs, repeat variable di-residues. (**d**) TALE-TFs can also either activate or repress transcription. (**e**) Schematic representation of the LITE-system (light-inducible transcriptional effectors) consists of DNA-binding TALE domain with the photosensitive protein CRY2 (TALE:CRY2) and CIB1 (interaction partner with CRY2), coupled with the desired effector (complex CIBI: effector). In the absence of light TALE:CRY2 are joined to the promoter region of a target gene, whereas a complex CIB1: effector remains free (OFF) (see the text). NLS, nuclear localization signal. (**f**) LITE system after light illumination, which confers conformational changes into the CRY2 protein, which subsequently recruits the CIB1:effector complex and a number of transcription factors to the promoter region of the target gene to activate transcription (ON) (see the text). (**g**) Schematic representation of the CRISPR/Cas9 system for genome editing, which consists of Cas9 nuclease domain and joined crRNA and tracrRNA for directing the Cas9 nuclease to the target site. The target site is indicated by scissors. PAM is shown inside the circles. (**h**) CRISPR/Cas9 system includes Cas9 nuclease domain and sgRNA for directing the Cas9 nuclease to the target site; (**i**) Site-specific binding of dCas9 with sgRNA can inhibit the interaction of TFs with a promoter region causing gene repression; (**k**) Site-specific binding dCas9:sgRNA fused to FD facilitates transcription

ZFNs (zinc-finger nucleases) genome-editing system utilizes chimeric proteins that consist of highly specific 'zinc finger' (ZF) DNA-binding domains fused to a nuclease domain of the restriction endonuclease FokI (Figure 2a). Each finger of the DNA binding domain, which consists of tandem Cys-His2 arrays, recognizes approximately three bp of DNA. Thus, a combination of six ZFs is sufficient to bind 18 bp of the unique target DNA sequence providing sufficient genomic specificity (Table 1). The nuclease domain can be substituted with other functional domains for manipulating the levels of gene expression (Figure 2b).

ZFN system has been successfully applied for the modification of various genomes, including plants,^7^ insects ^8^ *Danio rerio*,^9^ mice,^10^ rats,^11^ pigs,^12^ human cell lines^13^ and iPSCs.^13, 14, 15^

ZNF system showed promising results in gene therapy of the mutation causing sickle cell anemia in human iPSCs.^16^ Further, the bi-allelic correction of the point mutation (Glu342Lys) in the *α*1-antitrypsin gene (*A1AT* or *SERPINA1*) by ZNF system and subsequent re-introduction of differentiated iPSCs into the liver of the recipient mouse resulted in the restoration of structure and function of *A1AT* both *in vitro* and *in vivo*.^17^

#### TALENs and TALE-TFs

On the basis of the TALE protein TALENs genome-editing system has been successfully applied for genome modification in plants,^18^ insects,^19^ nematodes,^20^ the fish,^21^ amphibians,^22^ mice,^23^ rats,^24^ rabbits,^25^ cancer human cell lines, hESCs and iPSCs.^26, 27^ The most important component of this system is a site-specific DNA-binding protein TALE isolated from a pathogenic for plant organism *Xanthomona*s. Another TALE-like protein derived from a pathogenic bacterium *Ralstonia* can also be used for specific editing.^28^ The DNA-binding domain represents a TALE tandem repeats of 33–35 amino acids. TALE repeats have similar sequences and differ only in the two highly variable amino acids at positions 12 and 13 (RVDs, repeat variable di-residues), which form the basis for specific-nucleotide recognition.^29^ Four tandem repeats Asn-Asn, Asn-Ile, His-Asp and Asn-Gly are sufficient for recognition of guanine, adenine, cytosine and thymine, and, hence, for generation of TALEs with unique properties (see Table 1). The second element of this fusion is the nuclease domain of a restriction endonuclease FokI or another functional domain (e.g., VP64, TET1, KRAB, etc), which introduces specific changes to the genome (Figures 2c and d.).

Recently, a new variation of the TALE system, an optogenetic LITE system (light-inducible transcriptional effectors) has been developed.^30^ This LITE system consists of two components (Figures 2e and f). The first one is the DNA-binding TALE domain of *Xanthomonas* with the photosensitive protein CRY2 (TALE:CRY2) of *Arabidobsis thaliana*.^31^ The second component comprises CIB1 (interaction partner with CRY2), fused to a transcription activation domain (e.g., from a viral activator VP64), CIB1:TAD. In the absence of light TALE:CRY2 binds to the promoter region of a target gene, whereas the complex CIB1:TAD remains unbound (Figure 2e). The treatment of cells with light causes a conformational change to CRY2, facilitating the recruitment of the CIB1:TAD complex to induce transcription from the target promoter (Figure 2f). In addition to the regulation of transcriptional activity, such system can also be used for the targeting of specific epigenetic chromatin modifications to specific genomic loci.^30^

This approach allows studying the effect of selected chromatin modifications on the expression of specific genes. For example, the TALEs domain fused to the catalytic domain of TET1 protein (ten-eleven translocation), which oxidizes 5-methylcytosine to methylated cytosine (5 mC), was reported to cause a significant demethylation in the CpG-rich chromatin.^32^

#### CRISPR / Cas9 and dCas9-TF

The CRISPR/Cas system is a prokaryotic analog of the immune system against exogenous DNA-containing phages and plasmids.^33^ Although the exact mechanism of CRISPR/Cas9 action is still under investigation, it is deemed that clustered regularly interspaced short palindromic repeats (CRISPR) along with short spacer DNA fragments that derive from previous encounters with viruses are transcribed into long CRISPR RNAs (crRNAs). When combined with transactivation crRNA (tracrRNA), these crRNA:tracrRNA duplexes provide a 'search engine' for the Cas9 nuclease to attack specific viral DNAs^34^ (Figure 2g).

For the purpose of simplifying the implementation of the system in biotechnology, the crRNA: tracrRNA duplex was substituted with single-guide sgRNA^34^ (Figure 2h). The specificity of Cas9 homing is determined by the nuclease PAM motif and 20 nucleotides of the complementary sequence of sgRNA. PAM sequences vary among Cas orthologs: 5'-NGG-3 'PAM in *Streptococcus pyogenes*,^35^ 5′-NGGNG-3′ and 5′-NNAGAAW-3′PAM in *Streptococcus thermophiles*,^36, 37^ 5′-NNNNGATT-3′ PAM from *Neisseria meningitidis*.^38^ PAM dependence increases the specificity of CRISPR/Cas (Table 1).

The CRISPR/Cas system has successfully been applied for genome editing in plants,^39^ nematodes,^40^ insects,^41^ the fish,^42, 43^ mice,^44^ rat,^45^ human cell lines,^35^ ESCs and iPSC.^38, 46^

More recently, a new iCRISPR platform based on CRISPR/Cas and TALENs systems has been designed for quick (up to 1 months) and highly efficient production of bi-allelic knockout in hPSCs lines. First of all generation of hPSCs lines that express Cas9, the invariable component of the CRISPR/Cas system, was performed for creating such platform. For the next step the lipid-mediated transfection of small RNAs was determined as efficient for co-transfection of multiple gRNAs for multiplexed genome editing during a desirable stage of hPSC. To make the iCRISPR platform more flexible, special iCas9 hPSC lines were engineered for doxycycline-inducible expression of Cas9 through TALEN-mediated gene targeting. Thereby, this platform allows successful one-step generation of double- and triple-knockout hPSC lines as well as stage-specific inducible gene knockouts during differentiation of hPSC.^46^

Furthermore, the CRISPR system with dead Cas9 nuclease (dCas9) protein fused with a transcription activation domain (Figures 2i and k) has been developed. Activation and repression of specific genes in hPSCs thus affecting the course of differentiation has been achieved by employing this system.^47^

### Regulation of Oct4 expression and pluripotency

**Figure 3:**
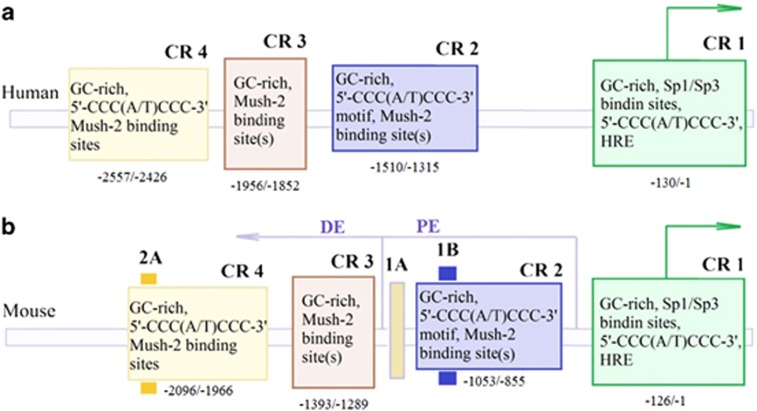
Structure of *Oct4* upstream promoter region. (**a**) Schematic representation of the *Oct4* upstream region of the human promoters.^52^ CR1-4 denote Conservative Regions in the promoter of *Oct4* gene (see the text). Conserved sequences are shown inside the boxes. Their locations relative to the start site are indicated below. Known transcription factors that bind these CRs are indicated. (**b**) Shown is the upstream region in the promoter of *Oct4* gene. Specific DE and PE sites with respect to the CRs are indicated.^52^ Green arrow denotes the direction of *Oct4* gene transcription

As was mentioned earlier, the activity of five transcriptional master-regulators is critical for the maintenance of pluripotency and self-renewal of stem cells. Among those, the *Oct4 (POU5F1)* gene is the critical one.^48, 49, 50, 51^ The Octamer-binding TF4 (Oct4) protein belongs to the family of homeodomain-containing transcription factors. Mechanistically, Oct4 not only positively affects transcription of genes required for pluripotency and self-renewal but also prevents the expression of TFs that drive differentiation of stem cells.^52^ The regulatory mechanisms of *Oct4* gene expression are quite complex. The *Oct4* gene is controlled by a TATA-less promoter (Figure 3), and two proximal and distal enhancers (PE and DE, respectively).^53^ There are four conservative regions (CRs) in the regulatory sequences of the *Oct4* gene: CR1, CR2, CR3 and CR4.^54^ CR1 (proximal promoter) and the most distal conserved region CR4 are the regions critically important for regulation of *Oct4* gene expression by several transcription factors, including Sp1 and RAR (Figure 3).^54^

Oct4 interacts with other TFs such as Sox2 and Nanog, which are also instrumental for the maintenance of pluripotency and iPSCs reprogramming,^55, 56^ thus forming a network of protein–protein interactions. As TFs exert their functions at least in part through the recruitment of epigenetic modifiers, it is not surprising that the promoter of *Oct4* gene is regulated by DNA methylation. Dnmt3a and Dnmt3b were shown responsible for DNA methylation of the *Oct4* promoter. This event is critical for triggering ESCs to differentiate.^57^ *Oct4* promoter is methylated and hence silenced in the vast majority of somatic cells. On the contrary, this gene is expressed not only in ESCs but also in several malignancies.^58^ For example, reactivation of *Oct4* is associated with tumor initiation in breast cancer cells^59^ as well as in poorly differentiated epithelial ovarian cancers.^60^ Exogenous delivery of specific cDNA combinations reactivates the endogenous *Oct4* promoter.

It needs to be mentioned that Oct4 is required not only for the maintenance of pluripotency, but when overexpressed it triggers differentiation. Thus, Oct4 serves a gauge of the cellular state in terms of commitment to differentiation.

### Application of ZF-TFs, TALE-TFs and dCas9-TFs systems in regulation of *OSKM* genes

#### ZF-TFs and expression of master regulators of pluripotency

Because the levels of Oct4 expression are critical for the fate of ES cells, it is not surprising that it is tempting to manipulate its levels by genome-editing tools.^61^ As mentioned earlier, ZF-TFs contain a zinc-finger DNA-binding domain and the functional domain to modulate gene expression. The ZF-TF system was successfully employed to target the *Oct4* gene expression. Specifically, ZFs targeting a 19-bp region between −25 and −7 bp downstream of the *Oct4* promoter were fused with either the herpes simplex virus VP16 activation domain or the repression domain from the human KOX1 protein.^61^ Transfection with the ZF-VP16 plasmid caused moderate, but reproducible activation of *Oct4*, whereas an overexpression of ZFs-KOX1 fusion caused a significant repression.^61^ Functionally, increasing or decreasing levels of *Oct4* expression by more than twofold forced ES cells to differentiate into primitive endoderm and mesoderm.^61^

ZFs attached to a Kruppel-associated Box (KRAB) domain function as potent transcriptional repressors via recruitment of the histone deacetylase (NuRD) complex, The latter includes histone deacetylases (HDACs), histone methyltransferase (SETDB1) and heterochromatin protein 1 (HP1).^62, 63^ Recently, almost complete repression of the *Sox2* gene via ZFs linked to a KRAB domain has been described in breast cancer cells.^64^ However, in addition to their well-established role as transcriptional repressors several KRAB-containing ZF chimeras can also activate transcription.^65^ For example, ectopic expression of KRAB-containing ZFs strongly reactivated *Oct4* expression in a panel of breast and ovarian cell lines.^66^ The KRAB domain is composed of two A and B boxes.

KRAB-associated protein 1 (KAB1) is one of the main co-repressors of KRAB and interacts with box A subsequently recruiting lysine methyltransferase SETDB1 to tri-methylate H3-K9 (H3K9me3).^67, 68^ Stabilization of the repressive complex on chromatin is maintained by binding of KAP1 with HP1 through interaction with H3K9me3. How the KRAB domain interacts with transcriptional co-activators is not known yet. One possibility is that KRAB–ZFs fusion may interfere with other transcriptional repressors (e.g., DNMTs), thus mediating the 'inhibition of inhibitors'. Irrespective of the exact mechanism, these results indicate that KRAB–ZFs can function as an activator of silenced genes in specific chromatin context.

ZF-TFs have been used for targeting of *Oct4, Sox2, Klf4* and *c-Myc* genes, which are critical for the maintenance and acquisition of pluripotency. The levels of activation for these genes were comparable to the ones observed in ES cells and did not require additional active epigenetic agents.^69^ Over 300 promoter region-targeting ZFs fused with the p65 subunit of NF-B were designed to target 1 kb (from –800 to +200 bp) region of the promoter around transcriptional start sites for each of *Oct4, Sox2, Klf4* and *c-Myc*. Each designed ZF-coding sequence was cloned between the N-terminal nuclear localization signal and the C-terminal NF-B p65 activation domain. To identify critical binding sites for ZF fusions to upregulate transcription, the upstream enhancer and the region downstream of the transcriptional start site were explored. Three out of the six constructs were shown by RT-PCR and western blotting to activate *Oct4* more than 16-fold in HEK293 cells. The best two of the three activator of *Oct4* are located in the upstream enhancer region of the gene. Also, the highest transcription activation of *Sox2* and *Oct4* in HEK293 was achieved with ZF-TFs containing VP64 or 2xp65 activation domains, respectively. However, the same ZFs fused to VP16 and VP64 domains failed to activate *Oct4* efficiently. Apparently, the ability of ZF-TFs to activate transcription depends on the cell type, the exact functional domain fused to ZF-TFs and the chromatin context of the targeted gene.^69^ In this respect, it can be speculated that depending on the epigenetic state of chromatin in the target locus, different functional domains fused to ZFs may exhibit various efficacies.^69^

#### TALE-TFs and master regulators of pluripotency

The TALE-TF system has successfully been employed to activate *Sox2* and *Klf4* genes in HEK293 cancer cell line.^70^ TALEs genome-editing system has also been used for reactivation of the *Oct4* gene.^71^ Designed TALEs efficiently upregulated *Oct4* transcription in ESCs, but failed to activate this gene in ESC-derived neural stem cells (NSCs) because of the repressive epigenetic state of the corresponding genomic locus. Chemical inhibition of histone deacetylases (HDAC) by VPA (valproic acid) and DNA methyltransferases by 5-azaC, respectively, greatly facilitated the effect of designed TALEs on expression of the epigenetically silenced *Oct4* promoter in NSC.^71^ This result suggests that designed TALEs can be used for reprogramming somatic cells into iPSCs.

TALE-VP64 fusion can induce transcription of endogenous *Oct4* by targeting its distal enhancer (DE). Reactivation of the endogenous *Oct4* by TALE-VP64 was sufficient for epigenetic reprogramming of fibroblasts into iPSC in the absence of exogenous factors Oct4 or Nanog.^72^ Mechanistically, TALE-VP64 likely recruited histone acetyltransferase (HAT) p300 to acetylate histones. In this respect, both TALE-VP64 and sgRNA/dCas-VP64 chimeras were shown to interact with p300 in human and mouse cells.^73^

Interestingly, TALE- and dCas9-based activators utilize different regulatory regions of the *Oct4* gene. The binding region from –120 to –80 bp was the most efficient for TALE-VP64-mediated activation, while Cas9d was highly effective when targeted by sgRNA to the region from –147 to –89 bp upstream of the transcription start site (Figure 3). In line with this, a significant increase of transcriptional activation of mouse *Oct4* promoters was achieved by moving the target sequences of inefficient TALE-VP64 into the –120 to –104 bp region.

Individual activators often exhibited marginal or no activity, whereas application of multiple TALE-VP64 or several sgRNA targeting the same region exhibited transcriptional synergy.^73^ Multiple TALE-VP64 targeting enhanced transcription of mouse *Oct4* gene up to 30-fold in NIH3T cells and increased transcription of the human *Oct4* up to 20-fold in HEK293T cells.^73^

#### dCas-TFs and master regulators of pluripotency

Recently, a CRISPRi (CRISPR inference) system has been utilized for regulation of transcription.^74^ In this system defective dCas9 lacking the nuclease activity was used. dCas9 when co-expressed with an appropriate sgRNA disrupts transcription by interfering with the binding or elongation of the RNA polymerase complex and/or specific transcription factors.^75^ This system provides means for transient attenuation of gene expression without causing deep epigenetic modifications to the DNA sequence.

Recently, a multiplexed activation of endogenous *Oct4, Sox2* and *Ilirn* genes by an inducible Tet-on CRISPR/dCas9 system has been developed for human and mouse cells.^76^ This system is based on the dCas9 protein fused to several copies of the viral transcription activation domain VP16. It was shown that dCas9-VP160 (10 copies of the VP16 minimal activation domain) efficiently activated endogenous genes when targeted by specific sgRNA to the region within 300 bp upstream of the transcriptional start site. The most efficient gene activation was achieved by clusters of 3-4 sgRNAs binding to the proximal promoters, suggesting a synergistic mode of action.^76^ Simultaneous induction of at least three different endogenous genes was achieved with the CRISPR-on system in this study.^76^

A recent report shed some light on the mechanistic differences in gene regulation by TALEs and Cas9d proteins. While TALE-TFs and CRISPR systems were comparable in their ability to repress transcription of endogenous *Oct4* and *Nanog* genes, TALE-TFs were much superior in their ability to activate transcription during the reprogramming of both MEFs and EpiSCs.^77^ Expression of Cas9d alone failed to reprogram cells into iPSCs despite modest upregulation of mRNA expression and positive effect in the luciferase reporter assay. It was likely due to an inefficient recruitment of p300 HAT to acetylate histones at the target site.^77^

In line with this notion, a recent report described a fusion construct between dCas9 and the histone acetyltransferase (HAT) domain of p300 as a powerful transcription activator.^78^ Specifically, the dCas9-p300 HAT protein targeted by a pool of gRNAs to the PE of *Oct4* gene 30-fold more potently activated transcription compared with dCas9-VP64. Moreover, this approach may be transferrable to other genome-editing systems (ZF-TFs, TALE-TFs), thus making it a versatile technology for targeted gene activation.

## Conclusions

Systems for genome editing and manipulation with gene expression based on DNA-binding ZFs, TALEs and CRISPR/Cas9 molecules fused to special functional or nuclease domain could be used in various areas of modern bioengineering. In particular, genome-editing systems represent a promising approach for generation of iPSCs (see Table 1 for comparison).

Importantly, genome-editing systems have a significant advantage over the existing OSKM scheme of generating iPSCs. The problem with OSKM is the induction of genomic instability and tumor formation especially by c-Myc and, to a lesser extent, Klf4. Comparative analysis of stem cells reprogrammed by expressing c-Myc revealed genomic deletions and amplifications, characteristic of oncogene-induced DNA replication stress.^79, 80^ One of the critical effectors of c-Myc overexpression is the major mammalian tumor suppressor TP53.^81^ TP53 is the guardian of genome protecting the organism from cancer as well as infertility or aging.^82, 83^

Among a large number of regulated genes, p53 activates expression of the *p21* gene, whose product, in turn, blocks proliferation and triggers differentiation of pluripotent cells.^84, 85^ To circumvent this problem several approaches have been described. For example, direct inactivation of p53 significantly increased the efficacy of iPSCs generation.^86^ Alternatively, inhibition of Notch signaling whose downstream target is p21 with small molecules also facilitated iPSCs generation.^87^ However, inactivation of p53 results in genomic instability and inactivation of Notch promotes differentiation of iPSCs into neural progenitors.^88^ In this respect, genome editing of downstream targets that prevent de-differentiation, for example, temporal inactivation of p21 or PUMA, would seem an ideal way to control these unwanted biological effects.^89^

Obviously, as any experimental system, genome-editing systems have their own limitations, that is, their efficacy varies greatly depending on the chromatin accessibility of a regulatory region selected for targeting, its proximity to the promoter or enhancer of the gene of interest, accessibility to other TFs for binding and so on. However, these obstacles will be avoidable in future once the working range and preferable epigenetic makeup of chromatin is determined for each particular targeting TF fusion.

Another potential limitation of this approach is based on the fact that most of the genome-editing systems fail to re-activate on their own epigenetically silenced genetic loci, such as *Oct4*. However, very recent data on epigenetic re-activation by targeting of the dCas9-p300 HAT domain fusion provide optimism on this end.^78^ Furthermore, there is a wealth of data arguing that isolated activation of the *Oct4* gene is not sufficient for generation of iPSCs. In this respect, it may be beneficial to combine the precision of genome-editing tools with a wider effect of pharmacological inhibitors. Future studies should test this intriguing possibility, which will then broaden the area of applications for the genome-editing tools.

## Figures and Tables

**Table 1:** A brief comparative summary of ZFPs, TALENs and CRISPR/Cas9 genome-editing systems

	**ZFPs**	**TALEs**	**CRISPR/Cas9**
DNA binding	'zinc-finger' domain	Transcription activator-like effectors (TALE)	crRNA:tracrRNA or sgRNA
Nuclease domain for genome editing	FokI	FokI	Cas9
Regulation of gene expression	ZF-TFs with VP16, VP64 and 2xp64 domains for *Oct4* activation; KRAB domain for *Oct4* both repression and activation.	TALEs with VPA and 5azadC inhibitors for upregulation of *Oct4*; *Oct4* enhancer targeting by TALE; VP64 for induction of *Oct4* transcription.	sgRNA/dCas-VP64 targeting for induction of *Oct4* transcription; dCas9 fused to VP160 and sgRNA for induction of *Oct4* transcription.
Efficiency	++	++	+++
Specifity	18–36 bp	30-36 bp	23–28 bp
Off-target	Vary	Low	Vary
Cytotoxity	Vary	Low	Low
The frequency of potential sites, limitations	1 to 100 bp. Limitation: absence of a collection of 64 zinc-fingers that would cover all possible combinations of triplets.	1 to 1 bp. Can be designed virtually for any DNA sequence. Limitation: the necessity of thymine at the 5'-end of the target sequence.	1 to 4–8 bp. Necessity of PAM sequence: 5'-X20 NGG-3', 5'-X20 NAG-3' or 5′-X20 NNNNGATT-3′.

## Abbreviations

CR: conservative region
CRISPR: clustered regularly interspaced short palindromic repeat
dCas9: dead Cas9 nuclease
DE: distal enhancer
ESC: embryonic stem cell
FD: functional domain
gRNA: guide RNA
iPSC: induced pluripotent stem cell
LITE: light-inducible transcriptional effector
OKSM: Oct4, Klf4, Sox2 and c-Myc
PAM: protospacer adjacent motif
PE: proximal enhancer
PSC: pluripotent stem cell
RVD: repeat variable di-residue
sgRNA: single-guide RNA
SL1-3: stem loop 1-3
TALE: transcription activator-like effector
TALEN: transcription activator-like effector nuclease
TF: transcription factor
ZF: zinc-finger protein
ZFN: zinc-finger nuclease
